# Supplementary material for: Developmental effect of RASopathy mutations on neuronal network activity on a chip
Source: Front Cell Neurosci. 2024 Jun 7;18:1388409. doi: 10.3389/fncel.2024.1388409 (PMC11190344; doi:10.3389/fncel.2024.1388409)
Supplement: Supplementary file 1 [file Presentation_1.pdf]

Supplementary Tables

Table S 1 Chemical reagents and antibodies

Reagent	Source	Identifier
Neurobasal^{TM} -A, minus phenol red	Gibco^{TM}, Thermo Fisher Scientific	Cat#12349-015
GlutaMAX^{TM} Supplement	Gibco^{TM}, Thermo Fisher Scientific	Cat#35050-038
B-27^{TM} Supplement (50X), serum free	Gibco^{TM}, Thermo Fisher Scientific	Cat#17504-044
Sodium Pyruvate (100 mM)	Gibco^{TM}, Thermo Fisher Scientific	Cat#11360-070;<br>CAS 113-24-6
Antibiotic-Antimycotic (100X)	Gibco^{TM}, Thermo Fisher Scientific	Cat#15240-062
HBSS without Mg/Ca (HBSS--)	Gibco^{TM}, Thermo Fisher Scientific	Cat#14175-129
Papain Dissociation System (PDS) Kit, EBSS Vial	Worthington Biochemical, Lakewood, NJ, USA	Cat# LK003188
Desoxyribonuclease I	Worthington Biochemical, Lakewood, NJ, USA	Cat# LS0002139
Dispase II	Supply Solutions Roche	Cat# 4942078001<br>CAS 42613-33-2
Poly-L-lysine hydrobromide	Sigma Aldrich	Cat# P1524<br>CAS 25988-63-0
(+)-Bicuculline	Tocris Bioscience, Bristol, UK	Cat# 0130/50<br>CAS 485-49-4
FCS (fetal bovine serum)	Sigma-Aldrich	Cat#F9665
Fluoroshield without DAPI	Sigma-Aldrich	Cat#F6182

Antibodies	Source	Dilution	Identifier
rabbit antibodies against VGAT cytoplasmic domain	Synaptic Systems	1:1000	RRID#AB_887869, Cat#131003
rabbit antibodies against VGLUT1	Synaptic Systems	1:1000	RRID#AB_887875, Cat#135303
Fluorescent secondary antibodies anti-rabbit Alexa Fluor 488	Jackson Immunoresearch	1:1000	RRID#AB_2313584, Cat#711-545-152
Fluorescent secondary antibodies anti guinea-pig Cy5	Jackson Immunoresearch	1:1000	RRID#AB_2340462, Cat#706-175-148

Table S 2 Features that were used in the principle component analysis and a brief description about their calculation

Feature	Description
mean firing rate (MFR)	Number of spikes detected in individual arrays during recording time divided by recording time
weighted mean firing rate to total (t) electrode number (wtMFR)	MFR weighted by the relation of currently active electrodes to number of electrodes (herein 16)
weighted mean firing rate (wMFR)	MFR weighted by the relation of currently active electrodes to number of active electrodes to reference point (e.g. time of maturity in time series)
interspike interval	Mean time between spikes in individual arrays during recording time
CV interspike interval	Coefficient of variance of ISI in individual arrays during recording time
Weighted mean bursting rate (wMBR)	The mean number of bursts in individual arrays per time weighted by the relation of currently active electrodes to number of active electrodes to reference point (e.g. time of maturity in time series), (Hz)
Burst duration	Mean duration of bursts calculated as the time from the first spikes to the last spike in individual arrays.
CV burst duration	Coefficient of variance of burst duration in individual arrays
Mean number of spikes per burst	The average number of within-burst spikes in individual arrays during recording time
CV number of spikes per burst	Coefficient of variance of number of within-burst spikes in individual arrays during recording time
Number of network bursts (NB)	Number of network bursts detected in individual arrays during recording time
Mean duration of NB	Mean duration of NB calculated as the time from the first spikes to the last spike in individual arrays.
CV duration of NB	Coefficient of variance of duration of NB
Mean number of spikes per NB	The average number of within-NB spikes in individual arrays during recording time
CV number of spikes per NB	Coefficient of variance of number of within-NB spikes in individual arrays during recording time
Mean number of contributing channels	Mean number of participating channels per NB in individual arrays during recording time
CV number of contributing channels	Coefficient of variance of number of participating channels per NB in individual arrays during recording time
Mean STTC	Mean values across STTC Matrix
CV STTC	Coefficient of variance across STTC Matrix
Skewness in STTC	Skewness in STTC values from STTC Matrix

Table S 3 Normality tests performed to test normality in the data sets of MEA-derived parameters; Four tests were conducted to lower the risk to fail in identifying the proper distribution. Here: 'no' indicates non-normal

**Data set**	**Statistical Test**			
	Anderson-Darling	D'agistion - Pearson	Shapiro-Wilk	Kolmogorov-Smirnov
wMFR	no	yes	no	no
interspike interval	no	no	no	no
wMBR	no	no	no	yes
Burst duration	no	no	no	no
No. of network bursts (NB)	no	no	no	no
Mean duration of NB	no	no	no	no
Mean no. of spikes p. NB	no	no	no	no
Mean STTC	yes	yes	yes	yes
Skewness in STTC	yes	yes	yes	yes

Table S 4 Identification of pathologically affected networks by test stimulus (STIM). Following steps were performed to select the valid wells by calculation of the relation between the evoked activity upon STIM2 and STIM1 and to check for outliers; According to web-page: Bhandari, P. (2022, November 11). How to Find Outliers | 4 Ways with Examples & Explanation. Scribbr. https://www.scribbr.com/statistics/outliers/ .

1. Calculate the relation between firing activity upon STIM2 and upon STIM1 for each well relSTIM = $\frac{evMFR_{norm}^{STIM2}}{evMFR_{norm}^{STIM1}}$
2. Calculate quartile 1 (Q1), quartile 3(Q3) and interquartile range (IQR) of relSTIM across all wells
3. Calculate upper and lower threshold for relSTIM and discard outliers

$$S^{upper} = Q3 + 1.5 \cdot IQR$$

$$S^{lower} = Q1 - 1.5 \cdot IQR$$

Table S 5. Overview of the datasets for the recordings of spontaneous activity of both genotypes (A: Ptpn11, B: Kras). The data show in detail the group size per experiment, and include information on the total number of recordings (# recordings), recordings that were excluded due to the exclusion criteria 3 (Table S 6) (#exclusions) and the total number of evaluated recordings (#evaluated).

**A: Sample sizes for spontaneous activity recordings of RASopathy model *Ptpn11*^{D61Y}**																		
**Control**	plate1				plate2				plate3				plate4				Total	
	#animals	#recordings	#excluded	#evaluated	#animals	#recordings	#excluded	#evaluated	#animals	#recordings	#excluded	#evaluated	#animals	#recordings	#excluded	#evaluated	#animals	#wells
DIV10-12	1	2	0	2	0	0	0	0	4	23	0	23	3	16	0	16	8	41
DIV15	1	2	0	2	0	0	0	0	4	23	0	23	3	16	0	16	8	41
DIV19	1	2	0	2	0	0	0	0	4	23	0	23	3	16	0	16	8	41
DIV21-23	1	2	0	2	4	18	0	18	4	23	0	23	3	16	0	16	12	59
DIV26	1	2	0	2	4	18	0	18	4	23	0	23	3	16	1	15	12	58
DIV29-30	1	2	0	2	4	18	0	18	4	23	1	22	3	16	2	14	12	56
**Ptpn11**	plate1				plate2				plate3				plate4				Total	
	#animals	#recordings	#excluded	#evaluated	#animals	#recordings	#excluded	#evaluated	#animals	#recordings	#excluded	#evaluated	#animals	#recordings	#excluded	#evaluated	#animals	#wells
DIV10-12	1	2	0	2	0	0	0	0	3	14	0	14	3	18	0	18	7	34
DIV15	1	2	0	2	0	0	0	0	3	14	0	14	3	18	0	18	7	34
DIV19	1	2	0	2	0	0	0	0	3	14	0	14	3	18	0	18	7	34
DIV21-23	1	2	0	2	2	9	0	9	3	14	0	14	3	18	0	18	9	43
DIV26	1	2	0	2	2	9	0	9	3	14	0	14	3	18	0	18	9	43
DIV29-30	1	2	0	2	2	9	0	9	3	14	0	14	3	18	4	14	9	39
**B: Sample sizes for spontaneous activity recordings of RASopathy model *Kras*^{V14I}**																		
**Control**	plate1				plate2				plate3				plate4				Total	
	#animals	#recordings	#excluded	#evaluated	#animals	#wells	#excluded	#evaluated	#animals	#wells	#excluded	#evaluated	#animals	#wells	#excluded	#evaluated	#animals	#evaluluatec
DIV10-12	3	14	0	14	3	14	0	14	3	21	0	21	3	7	1	6	12	55
DIV15/16	3	14	0	14	3	14	0	14	3	21	0	21					9	49
DIV17-19	3	14	0	14	3	14	0	14	3	21	0	21					9	49
DIV21-22	3	14	0	14	3	14	0	14	3	21	0	21	3	7	0	7	12	56
DIV24-25	3	14	1	13	3	14	0	14	3	21	0	21	3	7	0	7	12	55
DIV27/28					3	14	0	14					2	7	0	7	5	21
DIV30/31					2	14	2	12					2	7	0	7	4	19
**KrasV14I**	plate1				plate2				plate3				plate4				Total	
	#animals	#recordings	#excluded	#evaluated	#animals	#recordings	#excluded	#evaluated	#animals	#recordings	#excluded	#evaluated	#animals	#recordings	#excluded	#evaluated	#animals	#wells
DIV10-12	2	6	0	6	3	16	0	16	3	23	0	23	4	13	0	13	12	58
DIV15/16	2	6	0	6	3	16	0	16	3	23	0	23	4				12	45
DIV17-19	2	6	0	6	3	16	0	16	3	22	0	22	4				12	44
DIV21-22	2	6	0	6	3	16	0	16	3	22	0	22	4	13	0	13	12	57
DIV24-25	2	6	2	4	3	16	0	16	3	23	1	22	4	13	0	13	12	55
DIV27/28					3	16	0	16					4	13	0	13	7	29
DIV30/31					3	16	2	14					4	13	0	13	7	27

Table S 6: Note regarding the exclusion of data points:

**1.**	Selection of valid arrays through the MATLAB routine: A network on an array is categorized as active and further evaluated only when a minimum number of active electrodes (if not otherwise stated: 10) at DIV 21-24 is reached. An electrode is considered active with a minimum mean firing rate (MFR) of 0.1 Hz.
**2.**	Exclusion of whole MEA plate: In the experimental series conducted with KrasV model, on one plate a significant decline in activity (manifested as a decrease in MFR and the drop of number of active electrodes) was observed in a majority of arrays on DIV27/28 . Given the potential for systematic errors introduced by confounding factors, the plate was consequently excluded from statistical analysis starting from that time point.
**3.**	Exclusion of arrays due to loss of vitality: During the cultivation period, networks may suffer damage and experience a decline in vitality. This is reflected in a marked reduction in wMFR as well as a loss of active electrodes. In the arrays utilized, the activity at an electrode mirrors the neural activity of a group of neurons. A loss of active electrodes implies a substantial portion of the network has disintegrated. Networks are excluded only when both exclusion criteria are met: wMFR < 70%, and a drop of electrodes by at least 2.
**4.**	For the analysis of individual parameters assessing population-wide neuronal activity, the mean value across wells originating from a single animal was calculated. Consequently, animals with only one valid well were excluded from this animal-wise analysis. However, this rule was not applied to the well-wise analysis.

Supplementary Figures

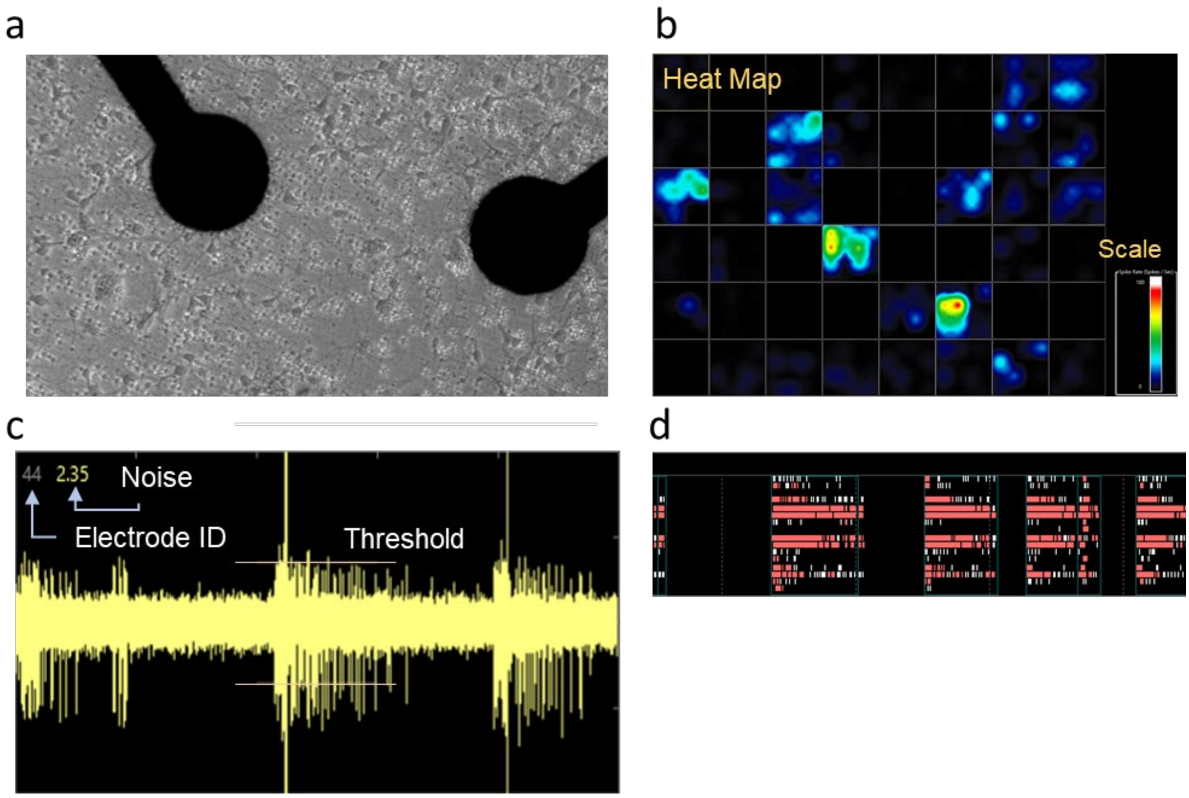

Fig. S 1 Visualization of cortical cultures derived from *Ptpn11*^{D61Y} mice and activity recordings in Axion Integrated Studio (AxIS) 2.4.2.

a Electrodes with neuronal culture on DIV 7 (scale bar: 400 µm); **b** Heat map visualizing network activity across the entire plate on DIV 21, one square corresponds to one well; **c** Continuous Waveform Plots displaying the continuous voltage recording for each electrode on DIV 21. Electrode ID and related noise level in µV are plotted. **d** Raster Plot indicating spikes on DIV 21; spikes within bursts are indicated as red strikes, network bursts are indicated as green rectangles.

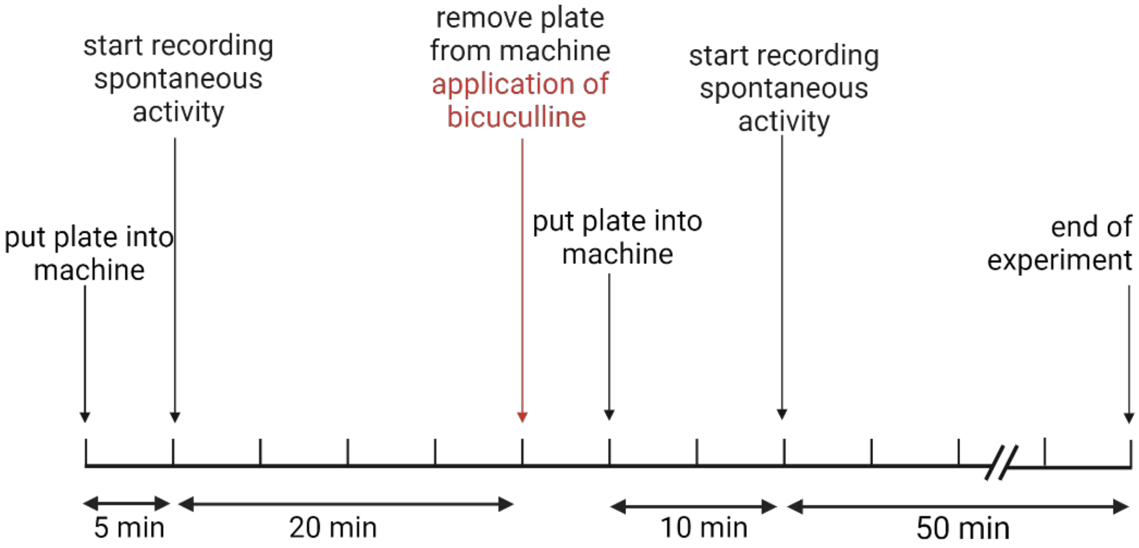

Fig. S 2 Experimental schema for testing the effect of disinhibition on neuronal activity in *Ptpn11*^{D61Y}. Individual steps of experiments are depicted on a timeline. Created with BioRender.com

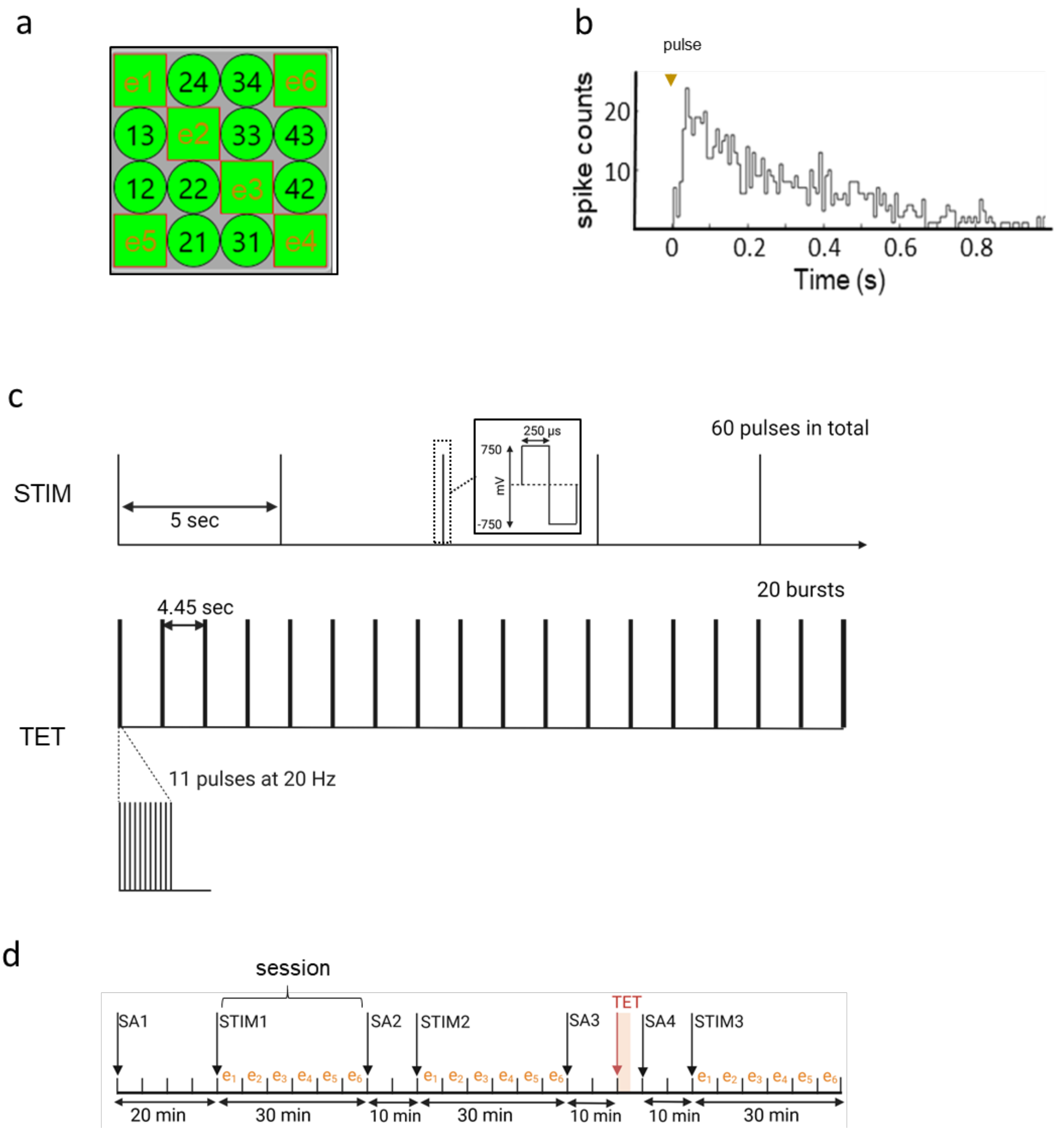

Fig. S 3: Experimental setting for recording of evoked activity in neuronal networks on MEA

a Schematic illustration of a multi-electrode array (MEA) indicating stimulation electrodes (e1-e6). The electrodes delivered test stimuli (STIM) subsequently (session 1-6) to record evoked activity. **b** Histogram shows number of spikes recorded upon electrical pulse with bin width of 8 ms and total time window of 1 s to sum up spike counts. **c** Stimulation protocols for test stimulus (STIM): 60 pulses at 0.2 Hz were applied to each of the six electrodes (resulting 5 min per electrode, 30 min in total) and for tetanus (TET): 20 bursts consisting of 11 pulses at 20ttzcc Hz were applied to one electrode; all pulses were delivered as biphasic pulse with characteristics depicted in the insert in c. **d** Time line of electrical stimulation protocol including recordings of spontaneous activity (SA) prior and after TET. **(c)** and **(d)** created with BioRender.com

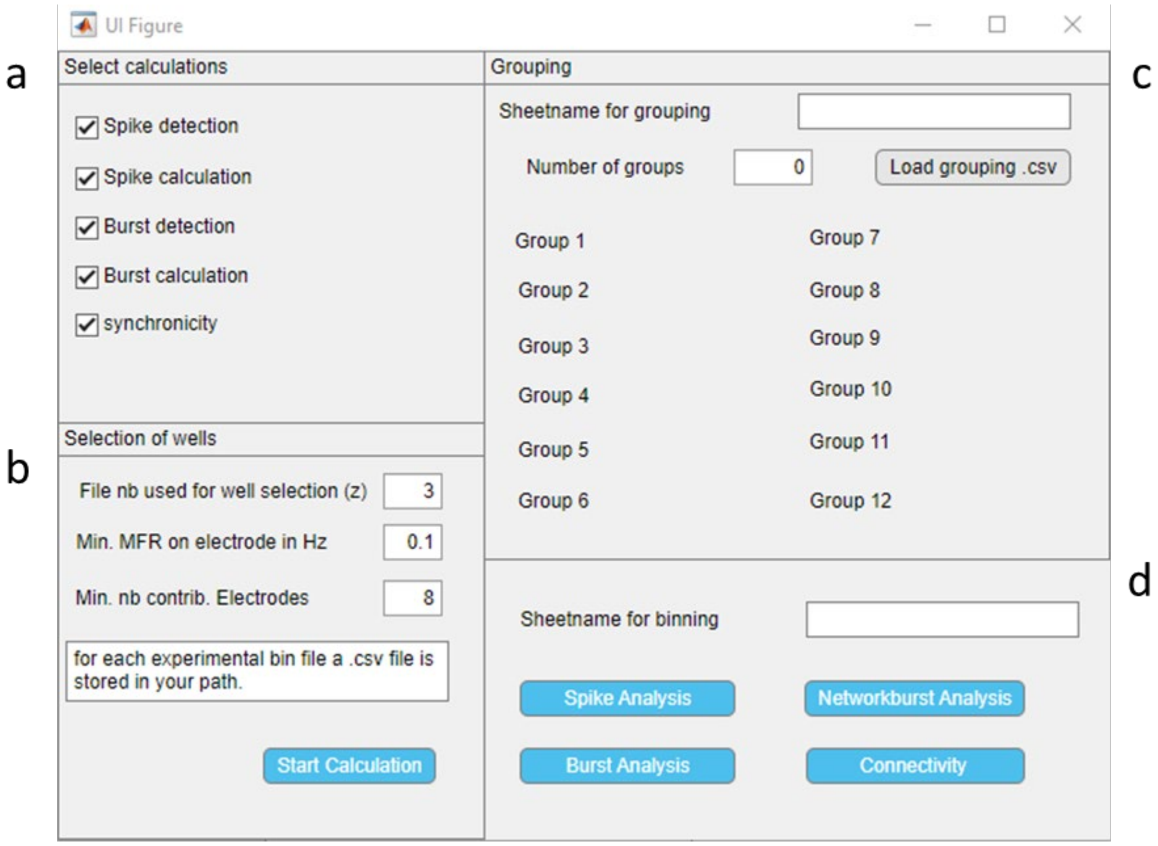

Fig. S 4 Graphical user interphase (GUI) of custom-written MATLAB based routine to analyse spontaneous network activity in neuronal networks grown on MEA.

a Check boxes for selection of operators; **b** Input field for the position (z) in the file list to determine recording/file to select valid wells, input field to determine inclusion criteria for contributing electrodes by minimum MFR (Hz) and for valid wells by minimum number of contributing electrodes per well; **c** Input field for the name of the excel sheet containing grouping information (sheet name for grouping); push bottom to select file and path of grouping file; input field for number of groups; input fields for group names. **d** Input field for name of excel sheet with information about averaging time units; push bottoms to initialize distinct analysis.

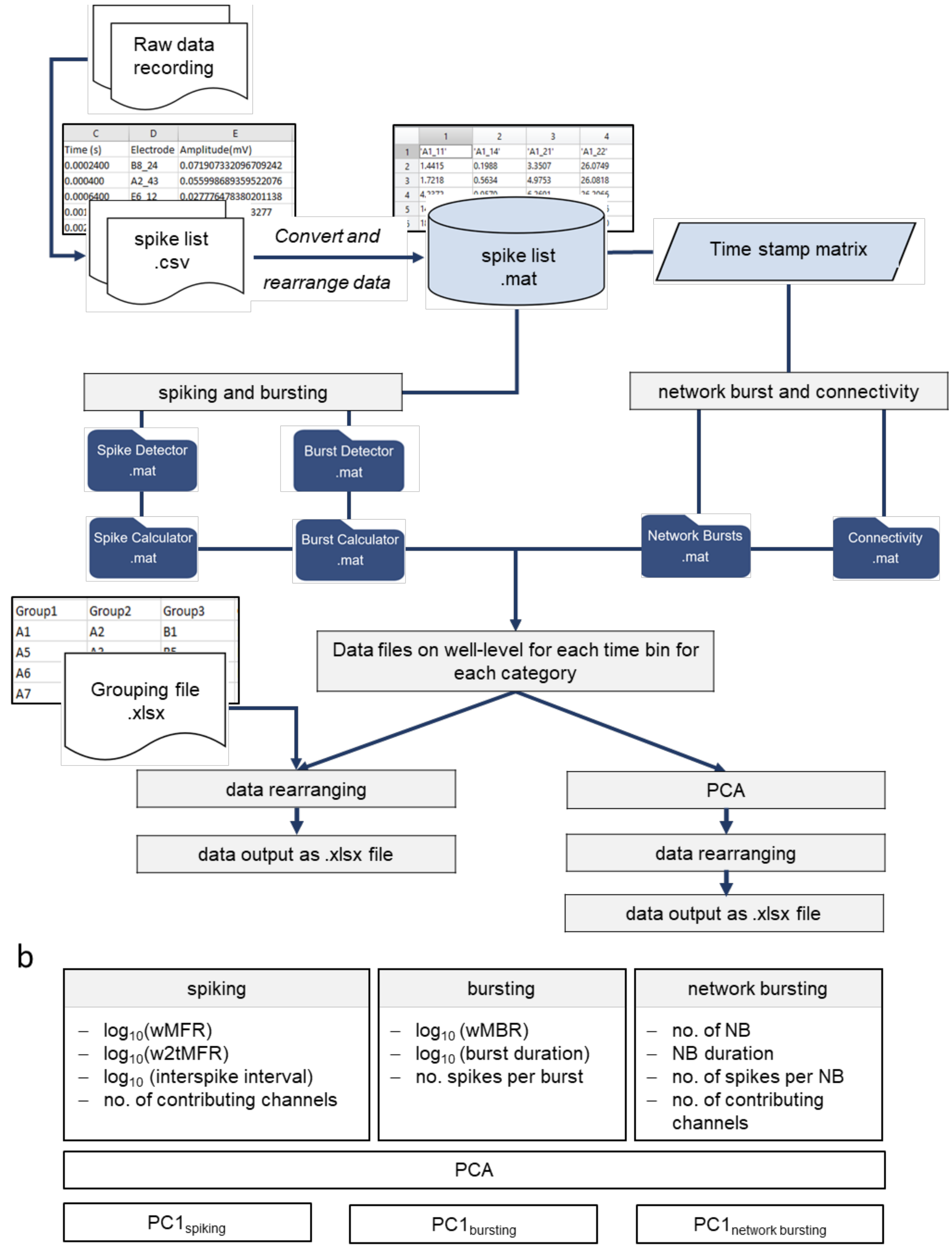

Fig. S 5 General schemes of data analysis procedures**.**

a Automatized analysis of spontaneous activity recordings in time series: raw data is converted to spike list files (.csv) in Axion Integrated Studio (AxIS) 2.4.2. Further processing is performed by custom-written MATLAB software package. Data set is converted and rearranged to a .mat cell array containing the time points of spikes and corresponding electrode name sorted in columns. Parameters describing spiking and bursting were calculated and output on well-level. For network bursting and connectivity parameters, time stamp matrix is calculated beforehand. Then, the parameters are either directly rearranged according to genotype, animal or treatment and averaged according to time units or principle component analysis (PCA) is switched in between. **b** The functional network features spiking, bursting and network bursting resulting from PCA dimension reduction projected on principle component (PC) 1.

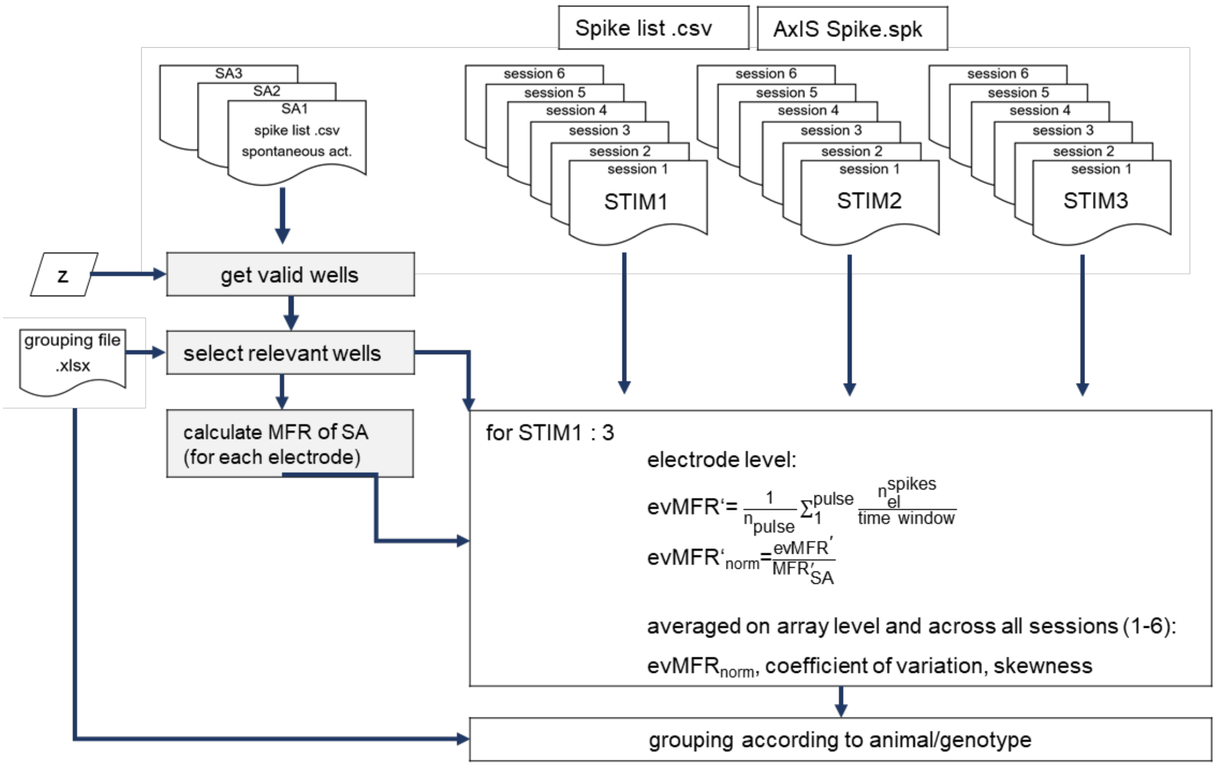

Fig. S 6 General scheme of automatized analysis of evoked activity by electrical stimulation.
Input data derives from Axion Integrated Studio (AxIS) 2.4.2. Input consists of .csv spike list files, containing information about electrode name and time point of each spike chronologically. Spike list files are entered from spontaneous activity recordings and evoked activity recordings, as well. Information about electrical stimulations is given in AxIS Spike .spk files. Data processing is performed by custom-written MATLAB functions. A session comprises triggering of six electrodes in a row with test pulses at 0.2 Hz for 5 min each. Selection of valid wells performs on a spontaneous activity (SA) recording prior to test stimulus (STIM) 1 defined by z (number of the related file in the folder containing all recordings) inserted by the user. Groups of wells are defined in the .xlsx grouping file. MFR from spontaneous activity recordings is used to normalize the evoked activity on each electrode.

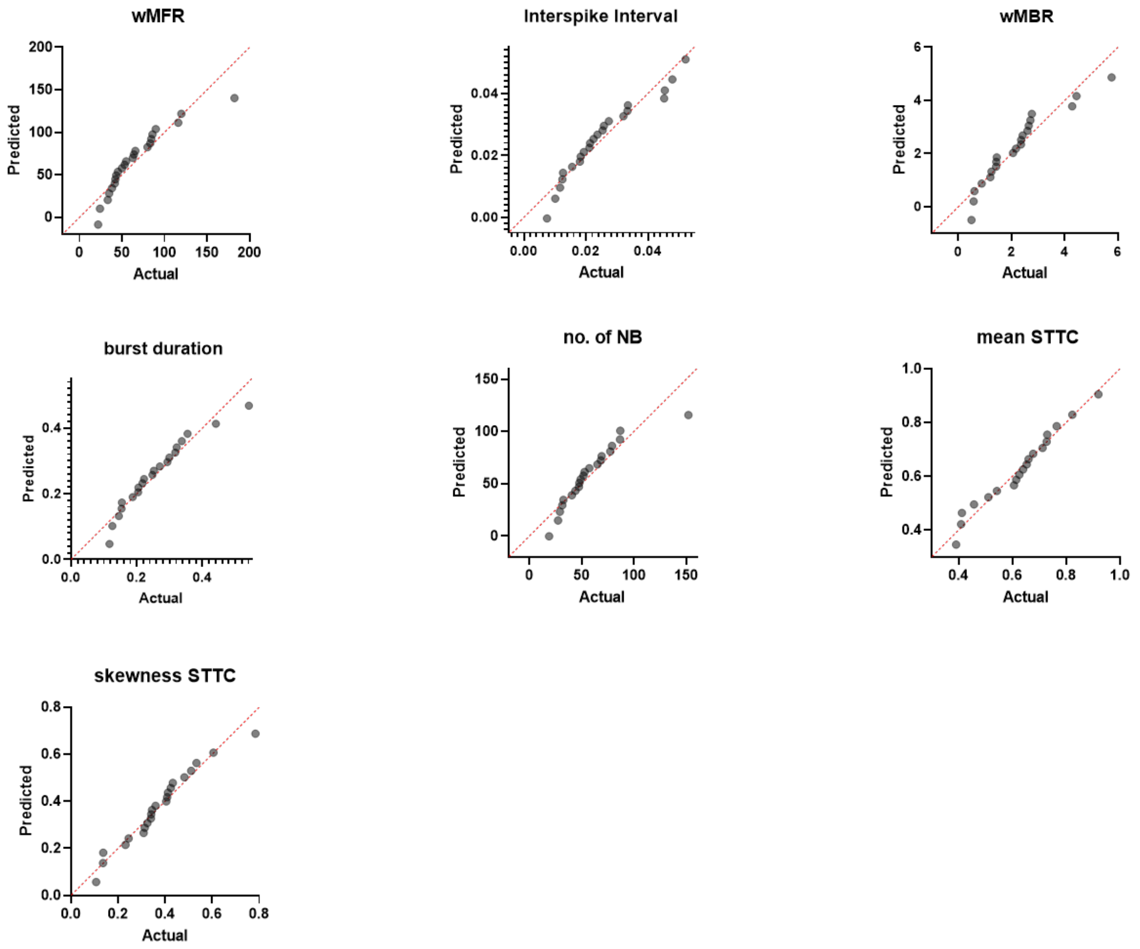

Fig. S 7 Distribution of data sets as normal QQ plots referred to distinct parameters from time series experiments derived from wild type and knock in of *Ptpn11*^{D61Y}.

QQ plots showing predicted residual versus actual residual. A data point represents the averaged value across the wells belonging to one preparation/animal on one plate (preparation level). Calculation is based on data recorded on DIV21.

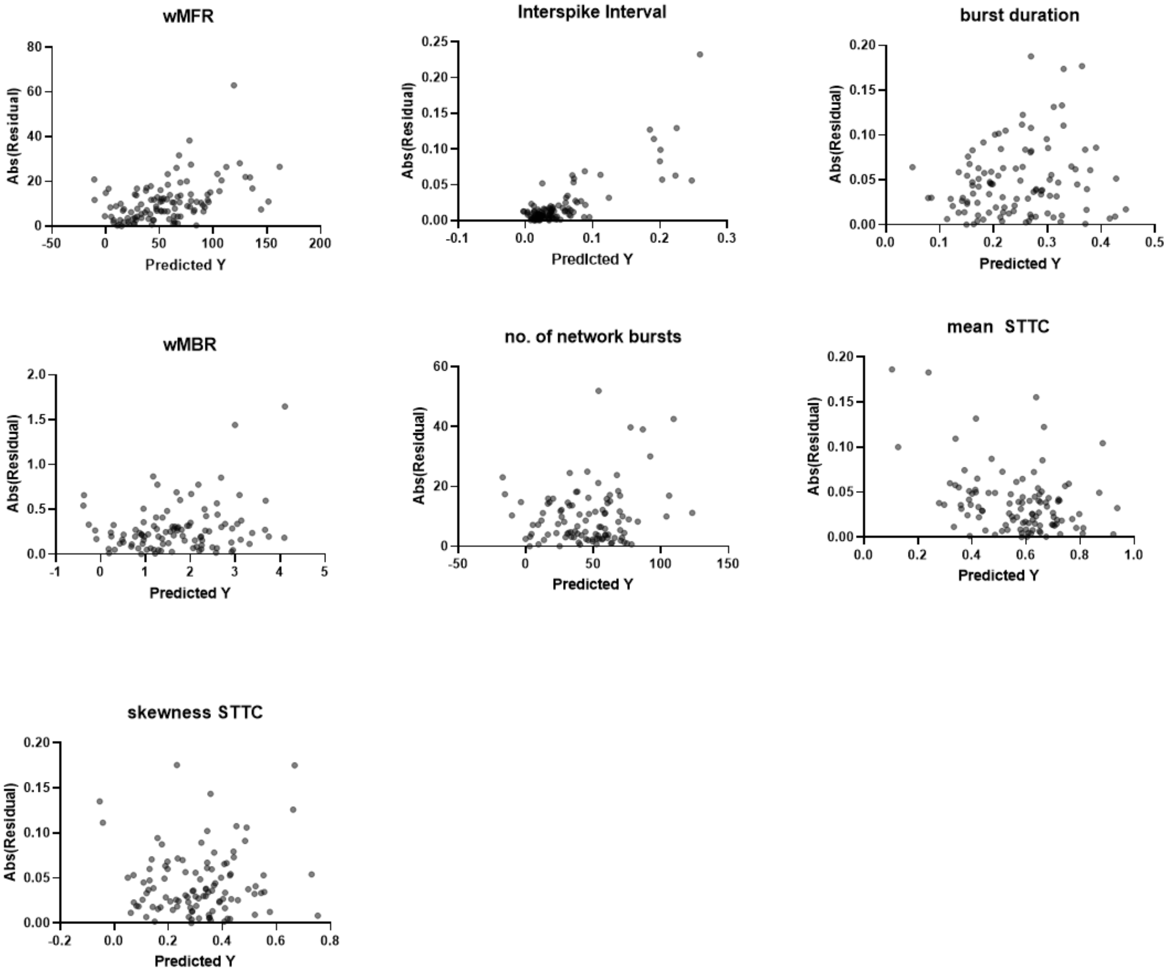

Fig. S 8 Homoscedasticity plots for recorded individual features describing spontaneous activity in longitudinal recordings in *Ptpn11*^{D61Y}.

Absolute values of residual versus predicted values of several parameter describing spontaneous network activity. A data point represents the averaged value across the wells belonging to one preparation/animal on one plate (preparation level). Calculation is based on the data recorded on DIV21.

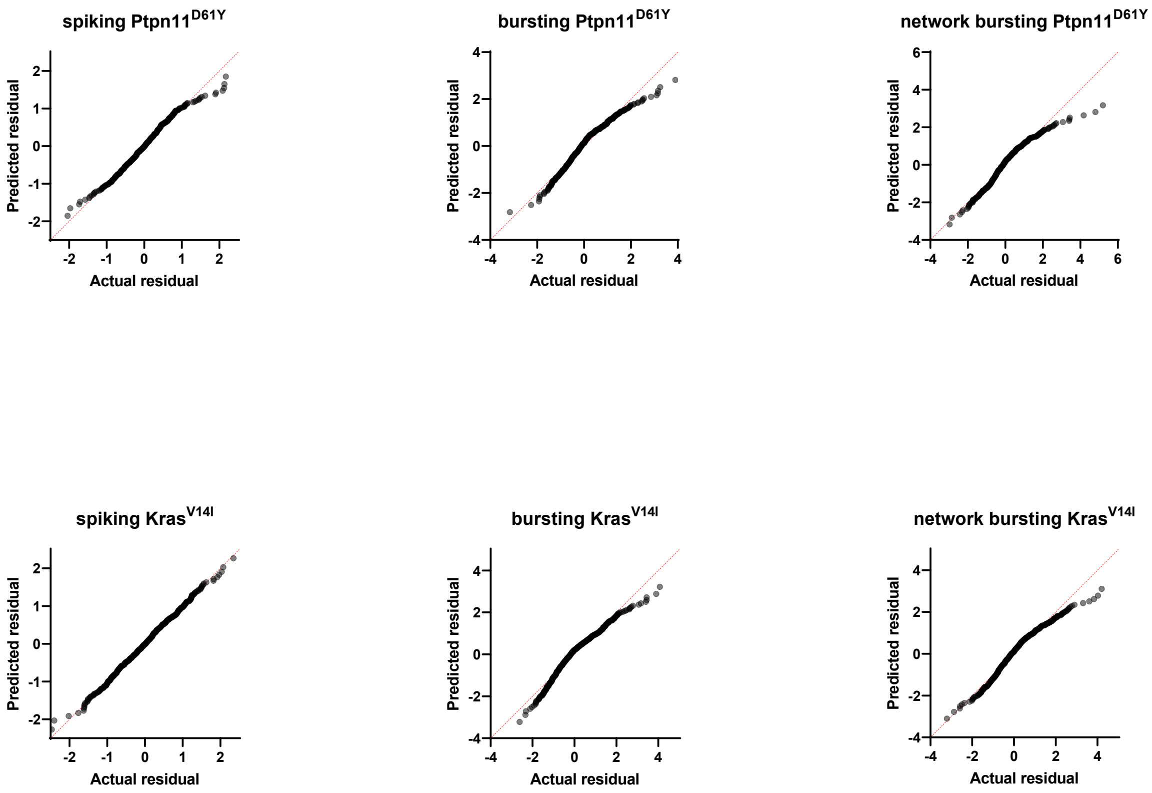

Fig. S 9 Distribution of PC1 projected data sets as normal QQ plots for *Ptpn11*^{D61Y} and *Kras*^{V14I}. Projection of feature vectors describing the fields spiking, bursting and network bursting onto principle component (PC) 1. A data point represents one well (well-level).

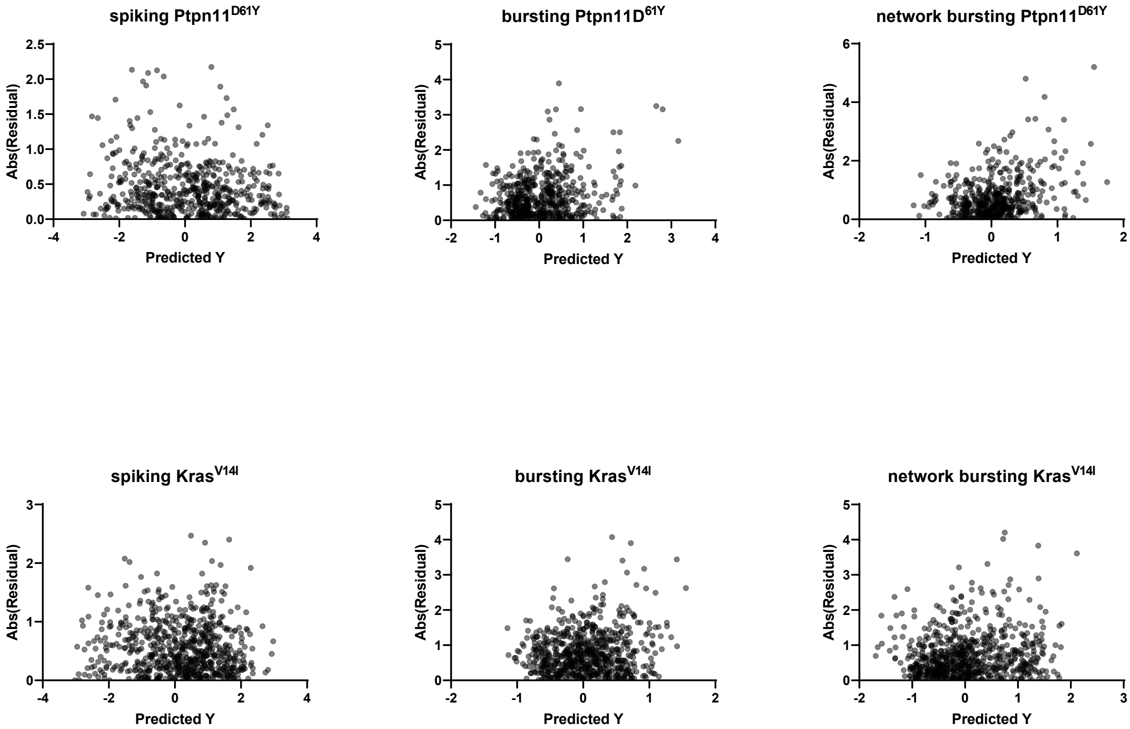

Fig. S 10 Homoscedasticity plots for recorded PC1 projected data sets for *Ptpn11*^{D61Y} and *Kras*^{V14I}.
Absolute value of residual versus predicted values of the projection of feature vectors describing the fields spiking, bursting and network bursting onto principle component (PC) 1. A data point represents one well (well-level).

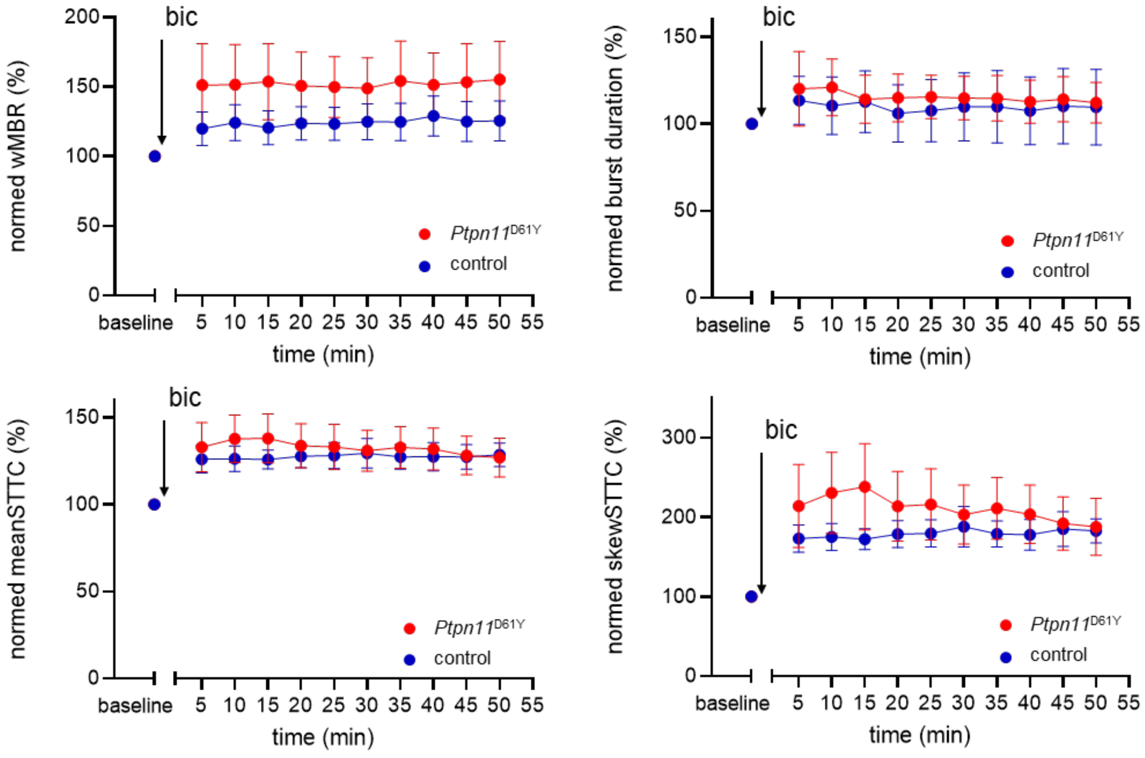

Fig. S 11 Effect of disinhibition on neuronal activity in *Ptpn11*$^{\mathrm{D61Y}}$ with time.

Line graphs with point representing mean ± SEM of several parameters describing network activity as a function of time prior and after application of bicuculline (bic) on DIV 33. Baseline values (averaged values over a time interval of 20 min prior bic treatment) were set as 100 %, values upon bic treatment were related to baseline.

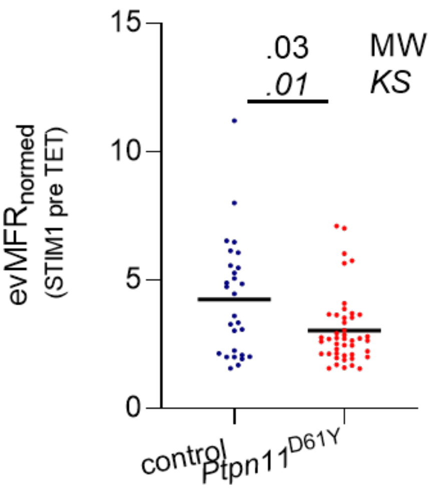

Fig. S 12 Assessment of evoked activity in *Ptpn11*^{D61Y} RASopathy model. Scatter dot plot demonstrating. evMFRnormed upon stimulation pre TET (STIM1), black lines indicate median values, one data point refers to one well. Significance was tested by Mann-Whitney (MW) to check differences in mean values and Kolmogorov-Smirnov (KS) to check differences in data distributions.

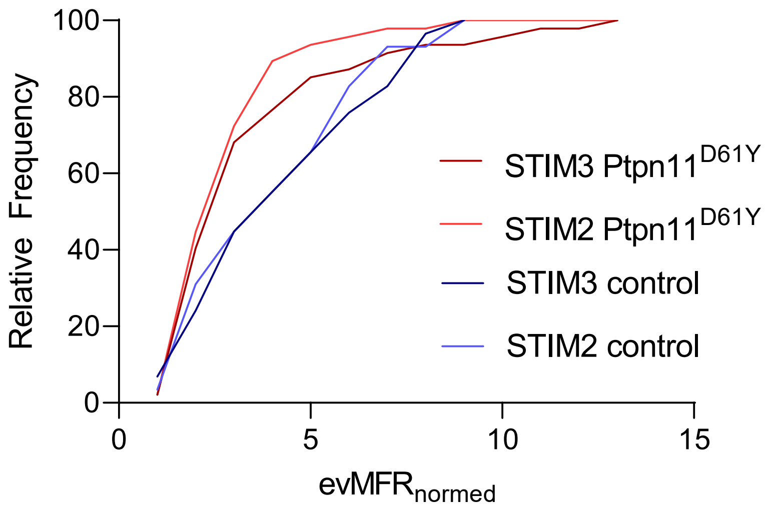

Fig. S 13 Relative frequency for evMFR$_{normed}$ upon STIM2 and STIM 3 for control and *Ptpn11*^{D61Y}
